# PreDictor Research in Obesity during Medical care - weight Loss in children and adolescents during an INpatient rehabilitation: rationale and design of the DROMLIN study

**DOI:** 10.1186/2050-2974-2-7

**Published:** 2014-03-10

**Authors:** Helene Sauer, Anna Krumm, Katja Weimer, Björn Horing, Nazar Mazurak, Marco D Gulewitsch, Frank Hellmond, Dirk Dammann, Walter Binder, Peter Linse, Stephan Zipfel, Stefan Ehehalt, Gerhard Binder, Aydin Demircioglu, Eric R Muth, Paul Enck, Isabelle Mack

**Affiliations:** 1 Department of Psychosomatic Medicine and Psychotherapy, University Medical Hospital, Tübingen, Germany; 2 Department of Psychology, Clinical Psychology and Psychotherapy, University of Tübingen, Tübingen, Germany; 3 Fachkliniken Wangen i.A., Children Rehabilitation Hospital for Respiratory Diseases, Allergies and Psychosomatics, Wangen i.A., Germany; 4 Public Health Department of Stuttgart, Department of Pediatrics, Dental Health Care, Health Promotion and Social Services, Stuttgart, Germany; 5 Department of Pediatric Endocrinology and Diabetology, University Children's Hospital, Tübingen, Germany; 6 Department of Psychology, Clemson University, Clemson, South Carolina, USA; 7 Department of Psychosomatic Medicine and Psychotherapy, University of Tübingen, Medical Hospital, Frondsbergstrasse 23, 72070 Tübingen, Germany

**Keywords:** Children, Obesity, Weight loss, Weight loss maintenance, Predictors, Inpatient, Rehabilitation

## Abstract

**Background:**

Obesity in adults and children is increasing worldwide at alarming rates. Obese children and adolescents are likely to become obese adults with increased risk of a number of comorbidities. In addition to preventing the development of obesity at young age, it is necessary to individualize the therapy of already obese children and adolescents in order to increase the likelihood of weight loss and maintenance. Therefore, the aim of this study is to identify predictors which play a significant role in successful weight loss and weight loss maintenance in children and adolescents.

**Methods/Design:**

Over a one year period, 60 obese children and adolescents between 9 to 17 years of age shall be recruited at an inpatient children rehabilitation facility in Germany. They will be investigated twice within a few days following admission and prior to discharge. The study will be an integrated component of an established inpatient weight-loss and in part psychosomatic therapy. The collected data can be grouped into four clusters: 1) demographic, sociometric and psychometric data, 2) objective and subjective parameters of body condition, 3) autonomic nervous system regulated functions and 4) objective and subjective parameters for eating behavior. Primary outcome is the change of the body mass index standard deviation score (BMI-SDS). In order to evaluate the data appropriately, all examinations will be also conducted in a normal-weight reference group, matched for age and gender.

**Discussion:**

For some of the collected parameters the time span between measures may be too short. Therefore, a 6 months, 1 year and 2 year follow-up will be performed for evaluating the different predictors and their influence in regard to a successful intervention. Further middle- and long-term follow-up studies are planned.

**Trial Registration:**

The study protocol was approved by the Ethics Committee of the University Hospital Tübingen, Germany. This study is registered at the German Clinical Trials Register (DRKS) with the clinical trial number DRKS00005122.

## Background

### Rationale of the study

Obesity and its associated effects are increasingly becoming a health problem worldwide – both in children and in adults [1]. In Germany – as in most other European countries – up to 15% of the children between 3 and 17 years are overweight and 6% are obese [2,3]. Obesity is characterized by an excessive increase in adipose tissue [4]. According to the World Health Organization (WHO) an individual is defined as overweight with a body mass index (BMI) ≥ 25 kg/m^2^ and as obese with a BMI ≥ 30 kg/m^2^[5]. However, these ranges are not transferable to children and adolescent values because of the different phases of life as a function of growth and puberty. Therefore, in pediatric medicine gender and age specific standard value tables are used for classification. By definition children and adolescents are overweight if their value is within the top ten percent and as obese if within the top three percent of their age group [5].

There are different inpatient and outpatient therapies to treat obesity in children and adolescents. These target changes in the fields of behavior, nutrition and physical activity [6]. In some cases, inpatient treatment is indicated, e.g. in children with severe obesity coupled with disturbed eating behaviors and psychosocial problems. For many children, these therapies are initially successful. In addition to weight reduction parameters like physical fitness, blood pressure, lipid and insulin levels as well as the psychological status improve [6]. After the intervention, it seems to be difficult for many children to maintain the skills they have acquired and to continue with weight reduction or stabilization at home, especially if no follow-up therapy is provided [6].

This raises the question which factors are crucial for the development of obesity, as well as which factors play significant roles in successful weight loss and weight loss maintenance in children and adolescents.

### Predictors of obesity, weight loss and weight loss maintenance

Different attempts have been made to identify potential predictors of weight loss and weight loss maintenance in the past [7-10]. Besides epigenetic and genetic approaches, these strategies can be subdivided into four clusters of investigations depending on the main focus of the researchers and research groups. These clusters include 1) demographic, psychometric and sociometric data, 2) objective and subjective parameters of physical condition, 3) autonomic nervous system regulated functions and 4) objective and subjective parameters of eating behavior. In most cases, interest has been limited to variables within one cluster rather than across clusters.

In addition, clinical variables such as actual weight, diagnosis, comorbidities [11], medication [12], metabolic status (e.g. fasting glucose levels and glucose tolerance) [13], and endocrine variables [14,15] have frequently been used to try to predict success of dietary management, but yielding little predictive ability. Often, a number of predictors are associated with obesity. Most often these include psychological, but rarely physiological [14], psycho-physiological [16] or neurocognitive functions [17]. Furthermore, few of these predictors have been studied for their predictive value of weight reduction and weight loss maintenance as part of inpatient or outpatient treatment.

#### Demographic, sociometric and psychometric predictors

It is known that in general the social environment of the child plays a crucial role in weight control [18,19]. This applies to aspects such as ethnic origin [20], income [21], health of parents [22] as well as their eating habits [23]. The EvAKuJ study showed - in a sample of 1916 overweight and obese children and adolescents - that in inpatient treatments only the BMI at admission was predictive of the short and long-term treatment success, whereas gender, all collected psychosocial variables and the parental BMI played no significant role [24].

#### Subjective and objective parameters for body condition

The total energy requirement of an organism is made up of the resting energy requirement, the activity dependent energy consumption, and food-induced thermogenesis [25]. This balance is substantially altered in adult obesity [26] and potentially in children as well [27].

An increase in physical fitness has a positive effect on body composition and weight, which has also been shown for children during an inpatient treatment for weight loss [28].

In childhood, sleep duration appears to have a significant impact on body composition. Gonnissen and colleagues (2013) showed an inverse correlation between changes in BMI and changes in sleep duration in puberty which resulted in neuroendocrine variations during adulthood. These variations were reflected in a positive energy balance with a coexistence of sleep disturbance [29]. Furthermore, it could be shown that increased sleep duration and an increase in physical activity were associated with positive changes in sleep behavior in inpatients during weight reduction [30].

It was recently shown in young people that parents, peers, media, and religious influences have an impact on the nature and extent of body satisfaction and influence the type and amount of consumed food and the frequency of physical activity [31-33]. Disturbances of body image are frequent in eating disorders [34] and also affect other parts of body awareness [35]. Sarisoy and colleagues (2012) compared obese individuals to healthy controls and found negative correlations between self-esteem, body image, and harm avoidance scores, and a positive correlation between body image and self-directedness in the obese population [36]. As yet, only few studies investigated the development of body image disturbances in children and report controversial results [37].

Disordered interoception (perception of intrinsic bodily sensations, e.g. hunger and satiety) has been reported as a secondary marker of diseases such as cancer cachexia and depression in the elderly. An interoception test (Schandry [38]) should allow investigating whether increased body weight and therapy changes are associated with bodily perception and whether these parameters influence the course of weight loss or regain.

#### Autonomic nervous system regulation

The autonomic nervous system (ANS) controls the maintenance of the balance of vital functions such as breathing, heart rate, and digestion and plays a crucial role in the regulation of acute and chronic stress responses [39]. BMI has a direct influence on heart rate variability (HRV) at rest and during exercise [40,41] and during fasting in healthy subjects [42]. Patients with obesity often show a changed balance between the (decrease) activity of the parasympathetic and the (increased) sympathetic nervous system [43] that normalizes after weight loss [44]. Another potentially relevant measure of ANS activity is the electrogastrogram (EGG) recording gastric myoelectrical activity [45,46] closely associated with cardiac HRV [47].

Psychosocial stressors such as parental divorce [48,49], poor mental health of parents [50,51] or of children themselves [52,53], are often associated with negative health outcomes [54-56], but appear to act as moderators rather than mediators of weight-loss. To our knowledge, the ANS response to acute (experimental) stress [57] has not been evaluated as a potential predictor of weight-loss and weight-loss maintenance in children and adolescents.

#### Subjective and objective measures of eating behavior

Smell and taste are often altered in patients with eating disorders and obesity, even in children [58,59], but data are controversial. It is not clear whether children who participate in weight loss programs have pathological sensory thresholds and whether these changes are normalized after treatment.

In obese children, hyperactivity of the limbic and paralimbic brain areas was observed during presentation of food stimuli in the hungry state. These areas remain activated after a meal and after the presentation of food stimuli [60]. It is not clear whether and how processing of food stimuli plays a role in weight loss treatment.

When subjects were asked to fill the same amount of water in a low, wide glass and in a tall, narrow one tend to fill more water in the low, wide glasses due to an optical illusion [61,62]. This implies that the estimation of food portion sizes may be affected by their visual appearance [61,62]. It is not known whether the number of correct/incorrect estimates of quantities of food and liquids is different in our study population and whether this has an impact on prediction of weight loss and weight maintenance.

An indirect measurement of eating behavior is the eating rate (run of a spoon or a fork to the mouth per time unit) [63]. Moreover, an increased eating rate may be associated with excess weight [64]. It was shown [65] that the movement of eating is different to other accidental or deliberate motor activities of the dominant hand or arm. On this basis, a device was developed called "bite-counter" [66] which can measure the eating rate. The bite-counter had not been evaluated in children and adolescents.

While the list of potential measurements within and across these clusters is virtually unlimited, their selection can be determined based on two criteria: a) feasibility in a pediatric inpatient setting at the beginning and the ending of a therapy within a limited time frame, and b) having been shown previously to bear relevance in (childhood and adolescent) obesity.

## Methods/Design

### Aims of the study

The aim of the study is to identify potential pre-treatment predictors of successful weight loss in obese children and adolescents during a standardized therapeutic treatment in an inpatient rehabilitation setting. In addition, repeated measures of the same variables at the end of treatment may demonstrate whether psychophysiological measures that respond to weight loss are associated with therapy success and whether they may serve to predict long-term weight-loss maintenance at follow-up. In addition, clinical parameters will be taken from the hospital charts and examined as potential predictor variables. We are aware that for some of the collected parameters the time span between measures may be too short. Therefore, follow-up studies are planned for evaluating the success of intervention.

### Study population

#### Patients and the inpatient setting

The obesity therapy concept at the Children Rehabilitation Hospital for Respiratory Diseases, Allergies and Psychosomatics, Wangen i.A., Germany is aimed at recovery from comorbid diseases associated with obesity, rediscovering joy of life, developing a forward-looking perspective, independent living, and growing up unencumbered. The children and young people are treated in accordance with the latest developments in medicine, especially pediatrics and in close cooperation with regional educational institutions such as the obesity academy (Adipositas-Akademie Baden-Württemberg e.V.), an atopic dermatitis academy (Neurodermitisakademie München/Wangen/Gaißach) and an asthma academy (Asthmaakademie Baden-Württemberg e.V.). Infants, children and adolescents are housed and cared for in small therapeutic groups with peers of the same age and in residential units situated on a park-like hospital ground. The clinic has its own kindergarten and a school with regular classes for all types of curricula.

Pediatricians, allergists, psychologists, social workers and nursing staff, teachers, trainers, dietetic professionals, occupational and speech therapists and staff for physical therapy work together as one team. The special obesity training is an integral part of therapy. The individual modules of the training are rooted in the concept of the clinic, including exercise and sports, change to a healthy diet, and weight-loss.

The nutrition concept is based on a balanced diet or “optimized mixed diet”. In nutrition training, patients meet weekly in small groups of 6–8 age-matched participants. In a training kitchen, all children and adolescents learn how to prepare food and cook with the aim of proper food handling and preparation when they return home. Additionally, they complete "shopping training" in a supermarket.

The hospital treats approximately 200 obese children and adolescents per year. The average duration of stay is 42 days.

#### Inclusion criteria

Children and adolescents with an indication for hospitalization for the purpose of weight loss - as set by their responsible primary or secondary care physicians - and with a BMI scoring higher than the 97th percentile specific for age and gender will be included [67]. The age range is set between 9 to 17 years. An indication for inpatient rehabilitation is given if the child suffers from diseases that are relevant or have negative impact on education, training or earning capacity (emotional, social development, personal development), especially for disorders/diseases that could have negative effects on the functioning of the particular age-appropriate levels of activity and social participation in all spheres of life. Medical rehabilitation is part of a long-term management of chronically ill children and adolescents and their families. The goal is an improvement, but at least to prevent deterioration (stabilization) of the health status-quo with regard to the current or future needs in education, training and profession. Requirement for inpatient rehabilitation is that the available outpatient options have been exploited and that a beneficial effect of the disease process can be expected.

#### Exclusion criteria

Children with severe psychiatric comorbidities, linguistic or intellectual limitations (by judgment of the treating physician or therapist), type-1 diabetes, tumors, systemic disorders, or severe cardiovascular diseases will be excluded.

#### Control group

A healthy normal weight reference group of 30 children with the same exclusion criteria will be recruited for potential predictor parameters for which age and gender-specific norm data are missing in the literature. These children will come from the catchment area of the University Hospital Tübingen, Germany.

### Outcomes

#### Primary outcome

The primary outcome measure is the change of the body mass index standard deviation score (BMI-SDS) [68]. SDS values indicate how many standard deviations an individual’s BMI lays above or below an age and gender matched median of BMI, a deviation of the value about two standard deviations above (+2) corresponds to the 97.7 percentile of the reference group [69]. Reference data for German children are used [67]. In order to calculate the BMI, the body weight and body height will be measured upon admission and upon discharge of their inpatient stay using calibrated instruments.

#### Secondary outcomes

Secondary outcomes measures include the changes in body composition and motor performance. Body composition is measured by a lipometer© (Möller Messtechnik, Graz, Austria) [70-72]. The motor performance is assessed by the Dordel-Koch-Test [73].

### Appointments

After informed consent, the hospital planning office will receive a screening form with psychophysiological assessments and will include the child into the study, depending on the particular time-slots available for the child. Afterwards, it will integrate four half-day assessments into the individual therapy plan. This procedure avoids time conflicts with other therapies and insures that the investigations are completed within the first week after admission and during the last week prior to discharge.

The children will pass two test sessions upon admission, one in the morning and one in the afternoon, and the same test sessions will be repeated prior to discharge. On both occasions, a series of investigations will be performed which can be grouped into four clusters: 1) demographic, sociometric and psychometric data 2) objective and subjective parameters of body condition, 3) autonomic nervous system regulated functions and 4) objective and subjective parameters for eating behavior (see below).

### Psychophysiological investigations

Within and across the discussed clusters, the number of measures chosen is based on theoretical (previous data available) as well as pragmatic reasons (feasibility, availability). For logistic reasons, the investigations are split into two groups, one of which is conducted during a morning session (9.00 h to 12.00 h), while the other one is conducted on a separate day in the afternoon (14.00 h to 16.00 h) (see Table 1). In addition, clinical data from the medical charts will be collected for the analysis. A list of all used questionnaires (of all clusters) is provided in Tables 2 (children) and 3 (parents).

**Table 1:** Measurement protocol

**Time a.m.**	**First session**	**Approximate duration**	**Time p.m.**	**Second session**	**Approximate duration**
**8:50**	**Welcome/general questions**	**5-10 min**	**14:30**	**Welcome**	**5-10 min**
	**Anthropometry**	**5-10 min**		**Media consumption questionnaire**	**5-10 min**
	**Sleep Self Report (SSR)/Pediatric Daytime Sleepiness Scale (PDSS)**	**7-10 min**		**Interoception test**	**5 min**
	**Taste test**	**5-10 min**		**ECG/EGG/SCL Baseline**	**20 min**
	**Inventory for recording the Quality of life in Children and Adolescents (ILK)**	**3-5 min**			
	**Smell test**	**15-20 min**		**Stress test**	**5 min**
	**Inventory of Eating and Weight problems Inventory for Children (EWI-C/IEG-K)**	**15-20 min**		**Post stress test**	**20 min**
	**Dordel- Koch test**	**15-20 min**			
	**Somatization Inventory for Children and Adolescents (SI-KJ)/State-Trait Anxiety Inventory (STAI-C)**	**5-10 min**		**Water load test**	**2-5 min**
				**Post water load**	**20 min**
	**Cognitive ability to discriminate volumes of food and liquids**	**15-20 min**		**Eating behavior test with snacks**	**20 min**
	**Depression Inventory for Children and Adolescents (DI-KJ)**	**10-15 min**			
	**Body image**	**10-15 min**		**Stress and Stress Coping Questionnaire for Children and Adolescents (SSKJ-J)**	**15-20 min**
	**Self-esteem scale by Rosenberg**	**2-5 min**			
**11:30**	**Farewell**		**17:00**	**Farewell**	

**Table 2:** Questionnaires for children

**Questionnaires**	**Short description**
Sleep Self Report (SSR) [74,75]	28 items; three thematic areas; three answer options ranging from “common” to “uncommon”
Pediatric Daytime Sleepiness Scale (PDSS) [76]	32 items; six thematic areas; five Answer options ranging from “always” to “never”
Inventory for recording the Quality of life in Children and Adolescents (ILK) version for children [77]	10 items; seven thematic areas; five answer options ranging from “very good” to “very bad”
Inventory of Eating and Weight problems Inventory for Children (EWI-C/IEG-K) [78]	60 items; ten subscales; four answer options ranging from “not true at all” to “totally agree”
Somatization Inventory for Children and Adolescents (SI-KJ) [79,80]	35 items; list of symptoms; five answer options ranging from “not at all” to “much”
State-Trait Anxiety Inventory (STAI-C) [81,82]	20 items; two scales; three answer options ranging from “almost never” to “often”
Depression Inventory for Children and Adolescents (DI-KJ) [83]	26 items; queries gradations of symptoms; decision between three predefined answer alternative
Stress and Stress Coping Questionnaire for Children and Adolescents (SSJK) [84]	84 items; three subscales; five answer options ranging from “never” to “always”
Revised self-esteem scale by Rosenberg	10 items; total score from 10–40, the higher the score the higher the level of self-esteem; Four answer options ranging from “strongly disagree” to “strongly agree”

**Table 3:** Questionnaires for parents

**Questionnaires**	**Short description**
Pediatrics Sleep Habits Questionnaire (PSQ) [85]	22 items; three subscales; Answer options: Item 1 to 16: “yes“, “no”, “don’t know”; item 17 to 22: four options ranging from “does not apply” to “meets most of time”
Epworth Sleepiness Scale (ESS-E) [86,87]	8 items; total score from 0–24; four answer options ranging from “would never doze” to “dozing high probability”
Children’s Sleep Habits Questionnaire (CSHQ) [88,89]	33 items; eight subscales; three answer options ranging from “common” to “uncommon”
Subjective Strength and Weaknesses (SDQ) [90,91]	25 items; five subscales; three answer options ranging from “does not apply” to “clearly applicable”
Inventory for recording the Quality of life in Children and Adolescents (ILK) version for parents [77]	11 items; nine thematic areas; five answer options ranging from “very good” to “very bad”

#### Cluster 1: Sociometrics and psychometrics

Besides conventional sociodemographic data (family history and status, social status, education etc.) all children will complete the following psychometric questionnaires, usually in validated German translations: a health-related quality-of-life scale (ILK, children and adults) [77], a depression scale (DIKJ) [83], a somatization scale in dependence on the International Children’s Somatization Inventory (CSI/SI-KJ) [79,80], the trait anxiety scale (STAI-C) [81,82], screenings for emotional and behavioral problems (SDQ) [90,91], and the self-esteem scale according to Rosenberg [92].

#### Cluster 2: Physical condition measures

The children will complete the Sleep Self Report (SSR) [74,75] which records sleep problems, and the Pediatric Daytime Sleepiness Scale (PDSS) [76]. Parents will fill in the Pediatrics Sleep Habits Questionnaire (PSQ) [85], a screening tool for sleep behavior, the ESS-E [86,87] determining daytime sleepiness, and the Children’s Sleep Habits Questionnaire (CSHQ) [88,89] once at the beginning of the therapy.

The Dordel – Koch test [73] will be used to capture the basic functions and motor performances of children and adolescents. The test battery consists of lateral jumping back and forth (coordination under time pressure, speed), sit and reach (flexibility of the hips and lower spine), standing long jump (explosive strength of the lower extremity), sit-ups (strength of the abdominal muscles and hip flexors), standing on one leg (coordination with precision tasks), push-ups (strength of the shoulder, chest and arm muscles) and the 6-minute walk distance (aerobic endurance). Additionally, physical fitness will be recorded by a regular bicycle ergometry program supervised by specially trained staff during the regular course of therapy. Body composition will be recorded by a lipometer© (Möller Messtechnik, Graz, Austria) that enables a non-invasive, fast, accurate, and reliable measurement of the thickness of subcutaneous fat [70-72] including hip and waist circumference. Moreover, the method is evaluated by a data set of 1351 juveniles of which 101 were obese [93]. In addition, anthropometric measures of various body parts will be collected by tape, calipers, and a scale. Thoracic breadth and depth will be assessed in order to detect the type of physiques according to the “Metric-Index" [94].

The interoception test according to Schandry [38] will test the ability of the children to correctly perceive body-internal cardiac signals (heart beat) [47,95]. During four intervals of 25, 35, 45, and 55 seconds duration, separated by 30 second resting periods, an electrocardiogram (ECG) will be recorded and children shall count their own heartbeats. A start and stop cue signals beginning and end of the counting phases. Participants are not allowed to take their pulse or to attempt any other manipulation that can facilitate the detection of heartbeats. Following the stop signals, children will be asked to report the number of counted heartbeats that will be evaluated against the recorded heart rate. Furthermore, a test for perception of their own body image will be performed by means of estimation of size of their body (total, parts) in relation to various external objects and sensory cues. Standardized tests for chemosensitivity (smell, taste) using validated tools (Burghart Messtechnik GmbH, Wedel, Germany) [96,97] to measure their respective thresholds, identification and discrimination ability will be conducted.

#### Cluster 3: Autonomic regulation

An electrocardiogram (ECG) will be recorded for off-line analysis of heart-rate variability (HRV) during rest (20 min) and following a mental stress test (counting backwards in steps of 7 from 1000, 300, or 100, depending on the age and intellectual level of the children; 20 min). Afterwards, the children will be asked to rate the perceived stress level on a scale between one (absolutely not stressed) to six (absolutely stressed). The ECG is recorded for inter-beat intervals (IBI) allowing Fast Fourier Transformation of data to estimate heart rate variability (HRV) parameters (high and low frequency (HF, LF)).

At the same time, three surface electrodes placed over the upper abdomen will record myoelectrical activity from the stomach (EGG) [98] for off-line analysis. At the end of the session, a water loading test [99,100] will be performed to determine the response of the stomach to an intragastric strain associated with gastric filling: subjects will be asked to drink as much water as possible within 5 minutes until the feeling of fullness is reached. The water load test will be followed by a 20 minutes measurement of the EGG. EGG signals will be analyzed with a Fast Fourier Transformation (FFT) procedure. A frequency of 3 cycles per minute (cpm) is regarded as normal gastric activity and between 4 to 10 cpm as tachygastria. This allows calculating the percentage spectral power from the total range of myoelectric gastric activity and computing the ratio between the percentage of the normogastria and the tachygastria band. This ratio has repeatedly been described as an indicator for disturbed myoelectric gastric activity, for example in patients with gastroparesis [99], but also in obesity [46].

Skin conductance will be measured parallel to ECG and EGG by using two separate finger electrodes on the dominant hand. For ECG, EGG and skin conductance measurement, a portable system (3991/3-GPP BioLog, UFI Company, Morrow Bay, CA, USA) will be used. Furthermore, a questionnaire survey of experience of stress and coping in childhood (SSKJ 3–8) [84] is added to cluster three.

#### Cluster 4: Eating behavior

For psychological assessment of eating behavior the inventory of eating and weight problems for children (EWI-C) [78] will be used, in addition, also a specific questionnaire asking for their food preferences and regular eating behaviors at home.

To validate eating behavior as indicated in the questionnaire, children will be allowed a 20-minute post-stress recovery (see above) while watching a movie on TV and taking snacks of different caloric density ad libitum, with the experimenter not present during that time. However, unbeknown to the children the snack plate will be filmed during this period. In addition, food will be weighted before and after the session and consumed calories will be calculated. In addition, some of the children will carry a novel "bite-counter" (Bite Technologies, Pendleton, South Carolina, USA) [66,101] that registers the numbers of arm and hand movements typical for eating behavior. Video-recordings will be used to validate the bite counting technology, as well.

Furthermore, the cognitive ability to discriminate volumes of food and liquids will be tested. Therefore, pairs of glasses (e.g. low, wide glass versus a slim, tall glass), of plates and bowls with pre-defined amounts of water or uncooked lentils (representing food) will be presented. The children and adolescents will be asked to estimate the quantities of the provided amounts. They will also be asked to estimate the amount of food needed to satisfy normal hunger.

### Ethics and registration of the study

The study protocol was approved by the Ethics Committee of the medical faculty for the University Tübingen, Germany. This study is registered at the German Clinical Trials Register (DRKS) with the clinical trial number DRKS00005122. The DRKS is an approved primary register in the WHO network. Children and parents were informed about the study purpose during the admission interview by a physician or therapist and were asked to provide written consent prior to inclusion.

### Statistics

We expect measures to be highly correlated within each cluster, and some variables within each cluster to be more sensitive or predictive to weight-loss during treatment than others. These key variables have been used as predictors for sample size calculation.

Cluster 1: Key variable: self-esteem scale according to Rosenberg

Cluster 2: Key variable: agility/motor activity (Dordel-Koch test)

Cluster 3: Key variable: ECG/IBI response to stress

Cluster 4: Key variable: caloric intake per time unit

To calculate optimal sample size, G*Power 3.1.7 [102] was used. To analyze the main research question (prediction of weight loss maintenance by key variables), sample size was derived for r^2^ deviation from zero (F distribution, Linear multiple regressions: Fixed model, R^2^ deviation from zero). Correlations of r = .3 (medium effect size [103]) were assumed for predictor-outcome associations as well as the predictor‘s intercorrelation matrix, resulting in a squared multiple correlation of r^2^ = .189 and an f^2^ = .234. With a level of significance at alpha = .05 and a statistical power (1-beta) of .8, a sample size of 57 would be optimal to decide on the predictive quality of the model.

Statistical analysis will be conducted in two steps: variables within each cluster will be tested for their intercorrelation, their responsiveness to weight change, and for their ability to predict weight loss in a one-by-one fashion (step 1). In cases of high within-cluster intercorrelation (as predicted), a hierarchical model will be constructed with the most sensitive variable per cluster to be entered into a multivariate regression model (step 2).

### Follow-up

The obese children will be included in a voluntary web-based aftercare program. This pilot program provides a possibility of periodic input of the actual weight and a graphical feedback of weight development, a chat option with the administrator for special questions regarding weight development and nutrition, and information and links to self-aid and support.

Six months after discharge the children will be contacted by phone and questioned about weight development and about implementation of the learned tools during therapy (portion sizes, activity in everyday life, coping of stress). Twelve months after discharge, children will receive a questionnaire and a prepaid return envelope by mail with an additional form for weight verification that has to be filled-in by a local pharmacist or a physician. The effort is rewarded with a gift certificate. A two year follow-up, and further middle- and long-term follow-up studies are also planned.

## Discussion

In conclusion, this study will discover potential psychophysiological predictors which play a significant role in successful weight loss in obese children and adolescents during a standardized therapeutic treatment in an inpatient rehabilitation setting. Carefully planned short-, middle- and long-term follow-up studies will be conducted in order to find predictors of successful weight-loss maintenance in obese children and adolescents. Some of the analyzed parameters may be useful as screening instruments to adjust future therapeutic intervention to the needs of the child.

## Abbreviations

ANS: Autonomic nervous system; BMI: Body mass index; BMI-SDS: Body mass index standard deviation score; ECG: Electrocardiogram; EGG: Electrogastrogram; FFT: Fast Fourier Transformation; HRV: Heart rate variability; HF: High frequency; IBI: Inter-beat intervals; LF: Low frequency; SDS: Standard deviation score; WHO: World Health Organization.

## Competing interests

The authors declare that they have no competing interests.

## Authors’ contributions

HS: responsible for conception and design of the study and will perform it, will acquire and analyze the data, drafted the paper. AK: will perform of the study, will acquire and analyze the data. KW: responsible for acquisition and preparation of the electrogastrogram part, will analyze the respective data. BH: responsible for preparation of statistics and will support the analysis of data. NM: responsible for acquisition and preparation of the heart rate variability, skin conductance data and will analyze it. MDG: responsible for preparation of the psychometry, stress behavior in children data, will support the analysis and interpretation of these data. FH: responsible for preparation and acquisition of clinical eating behavior data at the FW. DD: pediatrician responsible for the clinical logistics, supervision and preparation of the study at the FW. WB: responsible for clinical logistics, supervision, conception, preparation and participant recruitment of the study at the FW. PL: responsible for acquisition and preparation of clinical physical activity data at the FW. SZ: responsible for design and funding of the study. SE: pediatrician responsible for preparation of the study and will support analysis and interpretation of the data. GB: pediatrician responsible for preparation of the study and will support analysis and interpretation of the data. AD: responsible for construction and implementation of the follow-up website. ERM: provides technology and will analyze parts of the Bite-Counter data, as native speaker responsible for English writing. PE: responsible for conception, design and funding of the study and will support analysis of the data and paper writing. IM: responsible for conception, funding, design and preparation of the study, will acquire and analyze data, drafted the paper. All authors: read and approved the final manuscript.

## Authors’ information

Study was designed by a multiprofessional team. The first and last author are part of the research department Psychosomatic Medicine and Psychotherapy, University Medical Hospital, Tübingen, Germany and are interested in obesity in children and adolescents. For this reason, they approached the Fachkliniken Wangen i.A. and the present study design was established.
